# Integrating Global Health Within Dental Education: Inter-University Collaboration for Scaling Up a Pilot Curriculum

**DOI:** 10.5334/aogh.3024

**Published:** 2020-09-03

**Authors:** Ana Lucia Seminario, Belle Chen, Jennifer Liu, Brittany Seymour

**Affiliations:** 1 School of Dentistry, University of Washington, US; 2 School of Public Health, University of Washington, US; 3 Timothy A. DeRouen Center for Global Oral Health, University of Washington, US; 4 Oral Health Policy and Epidemiology, School of Dental Medicine, Harvard University, US

## Abstract

**Background:**

New education programs are developing to improve global health awareness. Dental students have demonstrated interest in international settings but are largely unaware of global health topics. The Timothy A. DeRouen Center for Global Oral Health of the University of Washington (UW) and Harvard School of Dental Medicine expanded a competency-based global health curriculum (Global Health Starter Kit) by integrating it within the UW School of Dentistry (UW SOD) existing elective course “Global Oral Health” to undergraduates, pre-, and doctorate students from the UW SOD and Public Health. The study objective was to evaluate the curriculum effectiveness by assessing 1) Knowledge and Attitudes (survey), and 2) Didactic coursework (global trends, global goals, primary care, social determinants and risks, and ethics and sustainability).

**Methods:**

Eligibility included enrolled students with both pre- and post-assessments. Descriptive statistics were conducted to present demographic data. Significant changes on survey and didactic evaluations were analyzed with paired t-tests (p < 0.05).

**Findings:**

The population (N = 15) represented 88% of the class. All Knowledge categories had a significant increase (p < 0.05), except in the topic of tropical diseases. At baseline, Attitudes categories had high scores and did not significantly increase by the end of the course. Even though all Didactic categories improved, only Social Determinants and Risks showed a significant increase (p < 0.01).

**Conclusion:**

Competency-based global health learning can be implemented in the dental curriculum. While the study shows promising results, efforts to identify areas for improvement as well as considerations of the institution’s culture need to be assessed and addressed for each teaching cycle.

## Background

Combined, oral diseases are the most prevalent chronic disease in the world [1]. The Global Burden Study found that oral diseases accounted for 17 million years lived with disability (YLDs) worldwide and approximately 17,000 Disability-Adjusted Life Years (DALYs) [2]. As interest, funding, and mobilization of projects in global healthcare increase, new programs are developing in dental, medical, and pharmaceutical curriculums to address awareness of global health in the health professions [3,4,5,6,7,8,9,10]. In the dental field, globalization has expanded the opportunities to engage with the global community addressing population health [7,8]. While there has been a history of dental volunteer work abroad, with a paradigm shift towards preventive dental care, Global Oral Health seeks to create partnerships with worldwide communities to assist in strengthening oral health programs. A recent survey of dental students showed that 83% of respondents had an interest in volunteering in an international setting while in school, and 92% showed interest for after graduation [8], yet the majority were unaware of the World Health Organization’s (WHO) basic package of oral care, the role of existing programs and services, or research in global oral health [9]. Integrating competency-based global health into the dental curriculum will increase an understanding of the current global environment, raise awareness for the burden of oral disease, and create opportunities for collaboration and partnerships with existing programs, research, and communities. Additionally, increasing accessibility of global oral health knowledge to students will provide an opportunity for students to network, mirroring the reality of a multidisciplinary health workforce.

At the University of Washington (UW), the Department of Global Health was developed in 2006 as the core for the UW’s initiatives in global health education, research, and service. The UW Timothy A. DeRouen Center for Global Oral Health (2017) at the UW School of Dentistry (UWSOD) recognized the need for an elective course that will bring global health to dental and pre-dental students and disseminate oral health education to public health students. In 2018, UW and Harvard School of Dental Medicine’s (HSDM) Office of Global and Community Health started a collaboration that aimed at increasing global oral health courses at educational institutions. For the 2019 class, the *Global Oral Health* elective at the UWSOD was adapted from the competence-based Global Health Starter Kit curriculum, a project of HSDM and the Consortium of Universities for Global Health [10]. This framework curriculum was developed to meet the Global Oral Health Interest Group of the Consortium of Universities for Global Health (GOHIG-CUGH) competencies for dental students [11]. The background of the project and collaboration was discussed in a prior publication [12].

At the UWSOD, the new elective course *Global Oral Health* was offered in the Spring of 2019 to undergraduate, pre- and post-doctorate students from the Schools of Dentistry and Public Health. In addition to the new curriculum, the course provided “From Theory to Action” lectures. These sessions aimed at providing real-life examples of how global oral health projects are planned (grant application), set up (grant development), and analyzed (data collection and data analysis) using research methods. Designed in an interdisciplinary approach, the goal was to introduce global health inequities, the burden of oral diseases at country levels as well as practical examples of inter-professional collaboration between oral and global health to a diverse group of young students at the UW Schools of Public Health and Dentistry. Through pre- and post-assessments, the overall objective of this study was to pilot the effectiveness and acceptance of a new global oral health curriculum by assessing 1) educational outcomes related to knowledge and attitudes associated with global oral health and 2) changes in global health knowledge using formal test questions. We hypothesized that our elective class will significantly impact students’ knowledge and perspectives of the relevance of global oral health in their future professional path.

## Methods

This cross-sectional study was approved by the UW Ethics Committee (#STUDY00009713).

### Course description

The course was directed by a faculty member with appointments in both the UW School of Dentistry and UW School of Public Health. Learning outcomes for students by the end of the course were to: 1. Describe a) global health inequities, b) global goals and trends, c) burden of oral diseases, d) primary care, e) determinants and risks, and f) ethics and sustainability of global oral health projects; 2. Identify and describe factors related to the success of inter-professional collaboration at international levels, 3. Propose research opportunities for inter-professional collaboration among oral health and a broad range of speakers’ disciplines. For this UW-HSDM partnership, we evaluated the best approach on how to integrate the Global Health Starter Kit within the UW culture, which excels at interprofessional research collaboration. We decided the elective would have four areas:

- *Didactics from the already tested HSDM competence-based program.* Students were introduced to international health care systems and the social, political, cultural, behavior, and economic factors influencing them through weekly seminar format and group discussion. The course used case studies with interactive student participation to analyze the impacts of oral health on the public health system.
- *Supplemental videos.* We identified informative TEDTalks that complemented each of the didactic modules (one per module). These supplemental videos were part of the weekly homework assigned prior to each class.
- *From Theory to Action.* We reached out to our collaborators from the UW Schools of Dentistry, Public Health and Nursing. These guest lecturers provided real-life examples of international research collaboration within Schools.
- *Group project.* By the end of the course, students were assigned to small groups on a final project to research and create a research question (Population, Intervention, Control, and Outcome (PICO question) for existing partnerships established with the UWSOD. The project was then presented and interactively discussed by the class.

### Knowledge, attitudes and behaviors towards global oral health assessment

A survey questionnaire was administered before and after the course was completed. The pre-survey questionnaire assessed each student’s initial perception of knowledge (8 items) and attitudes (7 items) adapted from a knowledge, attitudes, and behaviors survey developed at the Duquesne University School of Pharmacy [13]. Knowledge perception and attitudes were assessed using five-level Likert-type items (1 = strongly disagree, 2 = disagree, 3 = neutral, 4 = agree, 5 = strongly agree). For the post-survey, items in both assessments were repeated. The survey was optional to the course with no bearing on formal grade assessments.

### Formal Exam

A formal exam using the recommended tests from the HSDM’s Global Health Starter Kit was administered prior to the first lecture, and after the course was completed. The exam component included a multiple-choice formal knowledge test including twenty questions in the categories of global trends (5 questions), global goals (3 questions), primary care (3 questions), social determinants and risks (6 questions), and ethics and sustainability (3 questions) [14]. These categories reflect each course meeting, which consisted of interactive lectures that covered demographics of oral diseases, global milestones and current goals, policy implications, sociocultural and biomedical models for health, and concepts of optimizing positive impacts for all involved in global work. Like the survey, items in the formal assessment were repeated at the end of the course and had no bearing on formal grade assessments.

### Data collection and statistical analysis

Surveys and formal exams were completed on paper and all data was entered into REDCap (Research Electronic Data Capture) tools hosted at the University of Washington. REDCap is a secure, web-based application designed to support data capture for research studies. For descriptive analysis, distributions of the sample population were examined with regards to sex and education level. Pre- and post-survey means were calculated to assess changes in students’ knowledge, attitudes, and behaviors towards global health, and paired t-tests were utilized to assess the significance of changes in pre-/post-surveys. Pre/post-exam score means were calculated for each category of the formal exam (global trends, global goals, primary care, social determinants and risks, and ethics and sustainability) and for the entire formal exam, and paired t-tests were utilized to assess for significant changes in pre/post-exam scores. Statistical significance was set at 5%, and R software version 3.6.0 was utilized for analyses (R Foundation for Statistical Computing, Vienna, Austria).

## Results

Our study population (N = 15) represented 88% of the class. The majority of students participating in the class were female (60%) and in graduate school (60%) (Table 1).

**Table 1:** Demographics of participating students.

Variable	N (%) 15 (100)

Gender	
Female	9 (60)
Male	6 (40)
**Education Level**	

Undergraduate	6 (40)
Dental Student	6 (40)
MPH student	1 (7)
Dental Resident	2 (13)

### Knowledge perceptions and attitudes

Items were scored on the five-level Likert test (Score 1: Strongly disagree; Score 5: Strongly agree) (Table 2). There was a significant increase in knowledge in all categories except in neglected tropical diseases, which had a pre-survey mean score of 2.43 and post-survey mean score of 3.08 (p = 0.11) (Table 2). We found that at baseline, Attitudes categories scored high and had no significant increase at the end of the course (Table 2). The category with the highest improvement in score was “belief that learning patient’s culture, religion, and beliefs is important to delivering care”, with an increase from 4.70 to 5.00 (p = 0.08) (Table 2).

**Table 2:** Knowledge perception, and attitudes regarding global health.

Survey	Pre-score mean (SD)	Post-score mean (SD)	Mean difference	p-value**

***Knowledge and perceptions***				
I feel knowledgeable regarding the general topic and goals of global health	3.21 (0.70)	4.15 (0.69)	1.08	**0.01**
I feel knowledgeable about other countries’ healthcare systems	2.64 (1.08)	3.46 (0.97)	0.92	**0.04**
I feel knowledgeable about neglected tropical diseases	2.43 (0.65)	3.08 (1.04)	0.58	0.11
I feel knowledgeable regarding current issues in global infectious disease	2.79 (0.89)	3.69 (0.85)	0.83	**0.02**
I feel knowledgeable regarding cultural influences on health	3.50 (0.76)	4.23 (0.60)	0.75	**0.01**
I feel knowledgeable regarding drug discovery on the global scale	2.21 (0.80)	3.00 (0.71)	0.67	**0.02**
I feel knowledgeable regarding how organizations provide volunteer clinical care abroad	2.86 (1.03)	4.08 (0.86)	1.08	**0.01**
I feel knowledgeable about roles/opportunities for dentists in global health	2.71 (0.91)	4.08 (0.76)	1.33	**<0.01**
***Attitudes***				
I believe that global health should be considered an important issue to all dentists	4.57 (0.85)	4.77 (0.44)	0.25	0.28
I believe that ‘thinking globally’ improves the quality of care I can deliver	4.57 (0.65)	4.85 (0.38)	0.33	0.10
I believe that an experience abroad improves a dentist’s perspective of patient care	4.50 (0.65)	4.77 (0.60)	0.42	0.10
I believe that I can be involved in global health initiatives without traveling abroad	4.43 (0.85)	4.77 (0.44)	0.42	0.05
I believe that health issues primarily affecting people outside of the USA are still relevant	4.71 (0.61)	5.00 (0.00)	0.33	0.10
I believe that learning about a patient’s culture, religion and beliefs is important to delivering care	4.79 (0.43)	5.00 (0.00)	0.25	0.08
I believe that there is much for the USA to learn about health from around the globe	4.86 (0.36)	5.00 (0.00)	0.17	0.17

* Scale: 1 = strongly disagree, 2 = disagree, 3 = neutral, 4 = agree, 5 = strongly agree. ** Calculated using paired t-tests.

### Formal Exam

**Figure 1:**
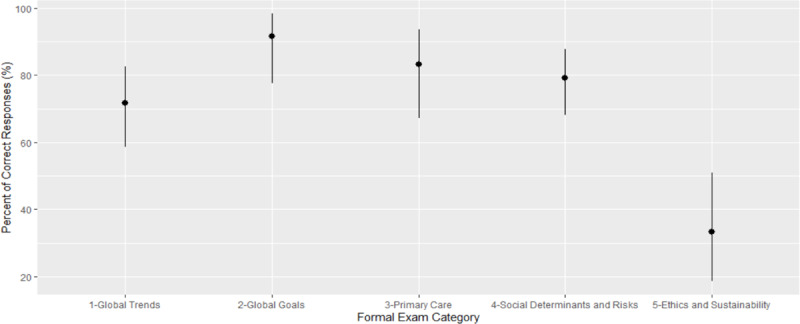
Percentage and 95% confidence intervals of accurate responses from all students for each category of the post-course formal exam.

From a maximum score of twenty, pre- and post-test score means were 11.56 and 14.58, respectively (p = 0.06) (Table 3). Although all categories improved in score, Social Determinants and Risks (0–6) was the only category with a significant increase (2.67 to 4.75) (p = <0.01). In the final exam, Global Goals had the highest percentage of correct responses (91.6%) while Ethics and Sustainability had the lowest (33.3%) (Figure 1).

**Table 3:** Formal exam from global oral health curriculum.

Global category	Pre-score mean (SD)	Post-score mean (SD)	Mean difference	P-value^a^

Global Trends*	2.57 (1.5)	3.58 (1.31)	0.91	0.10
Global Goals**	2.14 (0.77)	2.75 (0.45)	0.36	0.17
Primary Care**	2.07 (1.00)	2.50 (0.67)	0.09	0.72
Social Determinants and Risks***	2.67 (1.67)	4.75 (1.54)	2.30	**<0.01**
Ethics and Sustainability**	0.78 (0.67)	1.00 (1.013)	0.14	0.74
Total****	11.56 (2.46)	14.58 (3.18)	3.57	0.06

* Scores out of 5. ** Scores out of 3. *** Scores out of 6. **** Scores out of 20. ^a^ Calculated using Paired t-tests.

## Discussion

The purpose of this study was to pilot the feasibility and acceptance of the new *Global Oral Health* elective at the UWSOD by assessing 1) educational outcomes related to knowledge and attitudes associated with global oral health and 2) changes in global health knowledge using formal test questions. The topics for our learning objectives included goals of global health, other countries’ healthcare systems, current issues in global infectious diseases, cultural influences of health, drug discovery on the global scale, how organizations provide volunteer clinical care, and roles/opportunities for dentists in global health. We hypothesized that our elective class would significantly impact students’ knowledge perception of the relevance of global oral health in their future professional path. Results of this study support the effectiveness of integrating global health within the regular dental curricula.

While knowledge perception categories showed a significant increase between pre-and post-surveys, the change in attitudes was not significant. Specifically, the areas of no substantial improvement were a) belief that global health should be an important issue to all dentists, b) ‘thinking globally’ improves the quality of care, c) experience abroad improves a dentist’s perspective on patient care, d) one can be involved in global health initiatives without traveling abroad, e) health issues affecting people outside of the USA are still relevant, f) learning patient’s culture, religion, and beliefs is important to delivering care, and g) that there is much for the USA to learn about health from around the globe. The reason might be the initial assessment. The high baseline score informed us that our enrolled students were highly motivated and strong believers in the relevance of global oral health. Meanwhile in formal exam findings, we had an increase in scores overall and within each learning module. Yet, statistically significant improvement was observed only in the Social Determinants and Risks category. This relevant information allowed us to identify areas that will need not only a more in-depth instruction, but to provide additional material for the next teaching cycle. Improving formal test scores might require introducing service learning with self-reflection to critically apply didactic material. It is our plan to continue collecting data for the following 4 years that will allow us to provide trends for educational purposes.

While there are several publications on the HSDM Global Starter Kit [15,16], this is the first one that shows its potential for scalability by integrating it within an existing global oral health curriculum. Attention should be placed on assessing the institutional culture, strengths, and challenges for the merged curriculum to improve current efforts in dental education. In our case, specific changes for the 2019 class included a) quantitative analyses of the change on perceived and objective knowledge, b) weekly TEDTalks assignments, c) “Theory to Action” guest speakers, and d) a final project. Moving forward and based on the results of this manuscript, for the 2020 class we have added a) short videos that focuses on theoretical topics for each module, b) weekly Discussion Forums for students to provide qualitative feedback on weekly assignments, c) review of the tested questions at the end of each class. We will continue monitoring the learning curve and will conduct a qualitative analysis for the Discussion Forums and 5-year quantitative assessment of the impact of this elective course on our students’ professional development.

A review of the current literature in global health courses showed 4 common gaps in global health education: 1) they were frequently conducted in North American and European countries, 2) they were electives, 3) they were unstandardized, and 4) they targeted future medical professionals [17]. This study sought to close two of the gaps, by targeting an interdisciplinary cohort and by implementing a standardized curriculum designed for oral health. In Sweden, where four out of six medical schools have no global health education, students were surveyed at the end of their curriculum to find that they lacked knowledge and skills in the global burden of disease (51%), social determinants of health (52%), culture and health (60%), climate and health (62%), health promotion and disease prevention (66%), strategies for equal access to health care (69%) and global health care systems (72%) [18]. When comparing our results to other similar implementations of global health courses, we found similar results in a significant increase of knowledge, but no significant improvement in attitudes [12,19].

Study limitations include a small sample size that decreased statistical power and generalizability. Additionally, our knowledge, behaviors and attitudes survey was extrapolated from a pharmacy study. Yet, because the overall objective was to assess attitudes of health students on global health, we believe it was a good starting point for our project. Finally, students enrolled in the class had high interest in the topic and therefore scored high in attitudes. In order to better assess the impact of the course based on our current results, we will make efforts to have a more diverse student body for subsequent years. Specifically, we will use the results of this project to reach out to other units at the Schools of Dentistry and Public Health to make it part of the curricula for graduate students. We hope this course provides an appropriate framework to expose both dental and public health students to global oral health.

## Conclusion

This study showed that a) competency-based Global Health Starter Kit curriculum can be integrated in a global health elective course, and b) inter-university partnership, through integration of the Global Health Starter Kit project, can positively impact dental education by training our future leaders in global oral health. This process requires dedicated time to craft and adapt the validated program to the specific educational institutional context and learner needs. In addition, our study identified areas in our curriculum that need more depth in teaching and additional material. The sharing of the common curriculum between different institutional programs lays a foundation for progressing toward consensus building and promising practices for competency-based global health in dental education. Incorporating the topic of global health within the educational training of oral health researchers will enhance current international health agenda. While our study shows promising results, efforts to improve identified areas need to be assessed and addressed for each teaching cycle.
